# Piglet Viability: A Review of Identification and Pre-Weaning Management Strategies

**DOI:** 10.3390/ani11102902

**Published:** 2021-10-06

**Authors:** Bryony S. Tucker, Jessica R. Craig, Rebecca S. Morrison, Robert J. Smits, Roy N. Kirkwood

**Affiliations:** 1 School of Animal and Veterinary Sciences, The University of Adelaide, Roseworthy, SA 5371, Australia; roy.kirkwood@adelaide.edu.au; 2 Research and Innovation, Rivalea Australia Pty Ltd., Corowa, NSW 2646, Australia; jcraig@rivalea.com.au (J.R.C.); rmorrison@rivalea.com.au (R.S.M.); 3 Research and Innovation, Australian Pork Limited, Barton, ACT 2600, Australia; rob.smits@australianpork.com.au

**Keywords:** piglet, viability, management, preweaning survival

## Abstract

**Simple Summary:**

Neonatal piglet viability is decreasing in concert with the selection for ever-greater numbers of piglets born per sow per year. Their survival depends on the early intervention and management strategies used by production staff. This paper will review current and novel methods used to identify these piglets, some of the factors affecting their viability, and management strategies commonly used within production systems to improve their survival.

**Abstract:**

Increased attention on the effects of the global push for a larger litter size has focused on the increased occurrence of piglets with decreased viability, which have lighter birthweights and a reduced ability to thrive in early life. To improve their odds of survival, interventions must be timely and targeted. This requires the early identification of low-viability pigs and appropriate strategies to manage them. Using novel measures such as abdominal circumference and crown to the rump length in conjunction with birth weight may provide an improved protocol for the identification of those at most risk of preweaning mortality. Further, identifying these at-risk piglets allows interventions to increase their colostrum intake and heat provisions shortly following birth. The appropriate management of the pre- and post-partum sows will improve the chances of decreasing the number of piglets born with lower viability. However, this outcome is constrained by limitations in resources such as technology and staffing. If these challenges can be overcome, it will allow for greater control and increased effectiveness in the implementation of current and new management strategies.

## 1. Introduction

**Figure 1:**
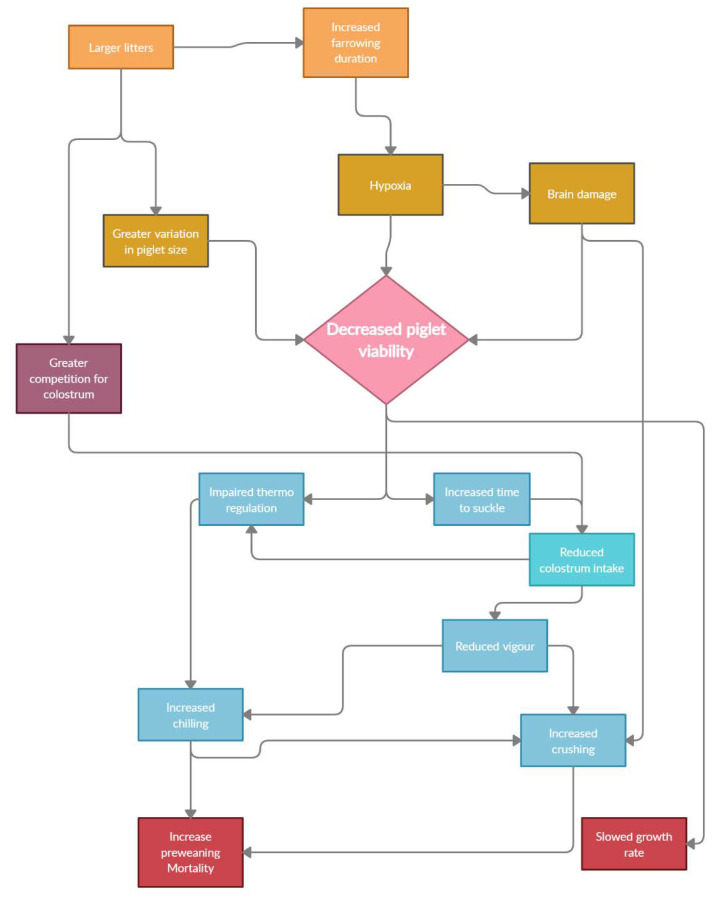
Factors influencing piglet viability and possible outcomes.

Pork industries worldwide face loss of profit due to high piglet pre-weaning mortality (PWM), which can account for 10 to 20% of all live born piglets [1,2]. Attempts to reduce PWM are ongoing through research and the formulation of new industry guidelines. However, this PWM continues to increase, with 80% of deaths occurring within the first 72 h of life [3]. There are many factors that impact PWM, although non-viable or low-viability piglets are a major contributor (Figure 1), second only to the occurrence of stillbirths [4]. The term viable, when referring to a foetus, is the ability to survive outside the uterus [5]. However, although the term viable, per se, is simple, the accuracy of measures used to determine the viability of piglets at birth is contentious. The absence of universally agreed measures for piglet viability contributes to the inaccurate prediction of survival and subsequent PWM. This raises concerns for the accuracy of identifying factors contributing to low viability and the effectiveness of associated remedial management strategies. Evidence within the literature strongly suggests that increased litter size, largely due to genetic selection to increase the number of piglets born, is the major contributing factor for the greater occurrence of low-viability piglets and the consequently increased PWM [6,7,8]. The continual increase in PWM indicates current methods to identify and manage low viability piglets are inadequate [9]. The aims of this review are to identify how the literature defines low-viability piglets and to evaluate the management strategies implemented to improve PWM. A variety of neonatal measures for viability, contributing factors and current management strategies will be discussed within this review (Figure 1).

## 2. Materials and Methods

### 2.1. Birth Weight (BW)

In commercial practice, BW is the most commonly used indicator of a piglet’s ability to survive until weaning [1,10,11,12,13,14]. The long-term effects of low BW on piglet survival and growth have been investigated, but with contradictory findings on the ability of piglets to compensate and catch up to littermates in terms of growth. It was documented that the lightest-BW piglets could compensate during postnatal growth [15] although others have stated that low-BW piglets have a higher chance of demonstrating poor pre-weaning growth and a lower BW at weaning [16]. Further, lower-BW piglets exhibit poorer lifetime growth rates and increased days to market weight compared to their heavier-BW littermates [6,15]. Low-BW piglets have been categorised based on their number of standard deviations (SDs) from the mean BW of the batch [11]. Within the experimental population of that study, piglets with a BW that was within 2.5 SDs of the mean BW of the population had the potential to compensate growth in other stages. However, piglets with a BW more than 2.5 SDs below the mean did not show compensatory growth. Regardless of the BW category, not all piglets showed improved performance, suggesting that the BW is not the sole predictor of pre-weaning growth and survival [11]. Some of the variation in performance may be the result of intrauterine growth restriction (IUGR). Many small foetuses are genetically small but are normal, while others are the result of placental insufficiency, often termed IUGR piglets. These IUGR neonates are a major contributor to perinatal mortality across many species. In humans, there are cut-off birth weights, and other morphological measures are used to identify IUGR babies [16]. There are two types of delayed foetal growth, asymmetric and symmetric. With asymmetric growth the body is disproportionately small for the head size, while with symmetric growth, heads are proportionally affected. The most commonly identified IUGR piglets are asymmetrical, identifiable by the so-called dolphin-like head shape [17,18]. These IUGR piglets grow at a slower rate than their littermates, with an impaired energy supply, underdeveloped organs and impaired gastrointestinal and skeletal development [19,20,21,22,23]. Symmetrical foetal growth restriction is harder to identify and, as such, possibly influences the performance reported for low-BW piglets in the literature. Only asymmetric IUGR is referred to in the literature for pigs, and the degree of IUGR severity can be identified by the use of morphological measures of the head [21,24]. However, the assessment of these head characteristics is subjective, and consequently, the identification of IUGR piglets using head morphology is often inconsistent. Thus, the validation of more objective measures of the head would improve the validity of classification. Head morphology is not the most robust method for identifying IUGR piglets. Clearly, the parameters used to classify piglets as having low viability require further clarification to improve the relevance and effectiveness of early intervention strategies for these low-viability piglets.

### 2.2. Novel Predictors

In humans, morphological measures have been recorded for newborns as being better indicators of potential growth and development than BW alone [16]. As such, the morphology of piglets at birth potentially may provide relatively novel predictors of their potential postnatal growth and development [18,21,23,25]. A variety of morphological measurements which improve the prediction of a piglet’s ability to survive have been identified. The body mass index (BMI = weight (kg)/crown – rump (cm)^2^) and the abdominal circumference (AC) have been identified as the most accurate predictors of piglet growth from days 1 to 28 of life, and thereafter, the AC and the ponderal index (PI = weight/crown – rump^3^) are the best predictors of growth from days 28 to 70 [25]. These findings are supported by Huting et al. [18], who found the BMI is associated with pre- and post-weaning average daily gain.

### 2.3. Colostrum Intake

Sufficient colostrum intake within the first 24 h of life is a well-established determinant for continued survival. Colostrum provides piglets with energy for thermoregulation and weight gain, passive immunity and growth factors [26,27,28]. It is recommended that to survive and thrive before and after weaning, a colostrum intake of at least 250 g per pig is required [29]. Accordingly, sufficient intake could be the most appropriate measure of viability. Amdi et al. [17] identified a large variation in stomach weight and capacity in both IUGR and normal piglets at birth. Under artificial feeding conditions, the maximum capacity of a newborn IUGR piglet’s stomach was ~50 mL per kg/BW [30]. This would require the feeding of colostrum at least 5 times within the first 24 h to achieve appropriate intake for improved survival. Piglet stomachs grow rapidly, and increases in functional maturation occur within the first 3 days post-partum [31], with gut closure occurring by about 24 h of life [27]. The rapid developmental changes in the stomach of the piglet highlights the importance of time relative to birth, if management strategies are to be implemented with optimal outcomes. However, applications within many production systems are currently impractical, as the sufficiently precise prediction of birth time is not possible, with approximately 15 h of the day (i.e., overnight) being unstaffed on most farms.

Regardless of the stomach capacity or frequency of feeding, recent studies have questioned the ability of the gastrointestinal tract of low-viability piglets to digest and absorb the colostrum components ingested [32,33]. The poorer nutrient absorption and potential lack of organ maturation in IUGR or low-viability piglets [17,34] could reduce the effectiveness of supplemental feed. Therefore, a measure of colostrum absorption requires research into the gut function of low-viability piglets as their ability to uptake colostrum is crucial. Intestinal closure is where the ability of intestinal cells to uptake macromolecules into the lymphatics and blood decreases. In the pig, this process begins at about first 6–12 h post-ingestion of colostrum and is complete at 24–36 h [35,36]. During the time when the gut is “open”, the piglet can obtain necessary immunoglobulins and other immune elements required to acquire their passive immunity [37]. This highlights the importance of timely intervention and the availability and uptake of sufficient colostrum. Additionally, when farrowings are unattended, a way to record the time of birth would aid in the timely implementation of effective management strategies.

### 2.4. Piglet Body Temperature

**Figure 2:**
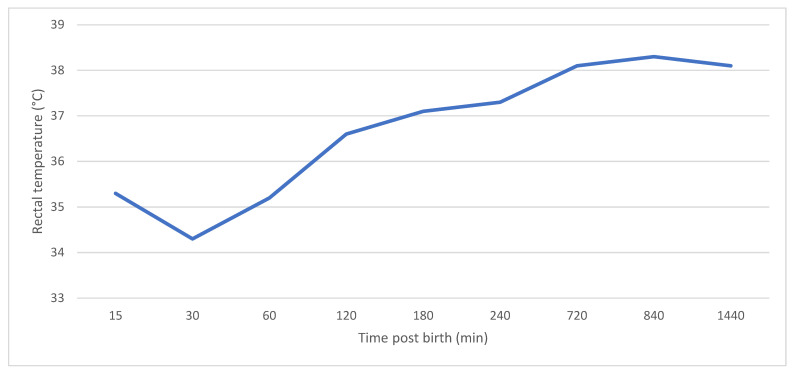
Piglet rectal temperature change from 15 min to 24 h post-partum. Modified from Anderson et al. [42].

Piglets are born with a limited energy supply, having little readily mobilizable adipose tissue and no brown fat that plays an important role in thermoregulation in many other species [38]. Piglets must rely on their ability to access a teat to suck and thermoregulate. The in utero temperature fluctuates between 38 and 40 °C [39] and is influenced by sow parity [40]. The minimum temperature of the environment immediately following birth must be 34–35 °C for thermoneutrality [41]. Ambient temperature varies greatly with the geographical location, and the temperature at the piglet level will be influenced by the type of production system. Commercial production facilities often operate with suboptimal environments for piglets, with draughts, skin wetness and cold flooring being contributing factors to PWM. This environment can directly impact the severity of the initial temperature drop and the time for piglets to recover to near the optimal temperature [42]. At their birth, farrowing house temperatures are often 10–12 °C lower than a piglet’s lower critical temperature of ~34 °C [39], accentuating reductions in piglet temperatures with near normal body temperatures not being achieved for several hours (Figure 2) [42]. Being born into a suboptimal environment could further negatively impact the chance of survival for a piglet already disadvantaged in utero. The ability of piglets to thermoregulate is directly related to their weight, and as such, their body temperature at 24 h post-partum may be a good predictor of early-lactation piglet performance [12,38]. Caldara et al. [43] showed that at 30 to 45 min post-partum, as weight increased, so did skin temperatures. This indicates less of a postnatal drop in body temperature of heavier piglets (≥1.4 kg) compared to that of lighter-weight piglets [44]. Further comparisons between weight groups were not reported, likely due to the small sample size (*n* = 4 sows) used in the study. Small piglets have a higher surface area-to-volume ratio than large piglets; thus, heat loss is proportionally greater. It has been suggested that a birth weight less than 1.1 kg in European breed piglets predisposes to an impaired ability to thermoregulate [39]. This may be explained by IUGR piglets usually being under 1.1 kg at birth and having a lower rectal temperature than normal-weight piglets [17,44]. There is potential for these measures to act as indicators for piglet survival in the immediate postnatal period and could influence decisions on the distribution of farrowing house resources and fostering movements.

## 3. Contributing Factors to Low Viability

### 3.1. Selection for Larger Litters

Many studies have focused on increasing the reproductive output of the sow, with increasing litter size at the forefront of industry research. Modern Danish sows have the capability to produce an average of 16.9 piglets born alive per litter [45], which is significantly greater than the available teat capacity. It is well established that larger litters at birth have a higher within-litter BW variation and a higher proportion of low-viability piglets [6,46]. This means that, despite higher numbers of piglets produced, piglets born alive are more likely to be relatively small, underdeveloped and at higher risk of mortality. The size of piglets from large litters is impaired in utero, because there is a greater competition for available resources [47,48]. The increased energy requirement of a large litter can also accentuate a lack of energy in the sow, contributing to an increased PWM due to difficulties during farrowing and inadequate colostrum and milk supply [49,50,51]. Extended farrowing durations as a result of a larger litter size can increase the incidence of intrapartum hypoxia, leading to higher stillbirth rates [52,53] and/or permanent brain damage in live born pigs (Figure 1) [54,55]. The increased occurrence of stillbirth is exacerbated in higher parity sows, as their uterine contractility may decrease, possibly due to older sows experiencing poorer calcium homeostasis, limiting the ability of the sow to expedite piglet delivery [56,57]. Although Australia lags behind many countries in terms of litter size, there is evidence that even with smaller litters there are trends in commercial herds for an increased total born alive per litter, with the Pork CRC reporting an increase of total piglets born from 12.4 to 12.8 between 2013 and 2016 and a similar trend also seen in the Australian average piglet PWM, being 10.9% and 11.5% for 2013 and 2016, respectively [58]. These reported figures indicate that although a slight increase in litter size was achieved, the net effect on overall productivity would be minimal given the higher mortality rate, as also described by others [59]. Accordingly, the increased presence of low-viability piglets in larger litters has encouraged the industry to develop better fostering and management techniques, but there are still a growing number of piglet deaths due to increased litter sizes and associated lower viability at birth.

### 3.2. Farrowing Induction

Induced farrowing is used to increase the likelihood of the sow farrowing during supervised hours [60,61]. Indeed, supervision is recommended when inducing farrowing, as several potential negative side effects have been reported [62,63]. Some studies report an increase in stillbirths, but this is largely considered a result of extended farrowing duration due to dystocia [52,63]. Regardless, if not timed correctly, inducing sows to farrow has been associated with an increase in PWM [62]. Premature piglets born as a result of inappropriately timed farrowing induction are born lighter and have a lower average daily gain than those born to non-induced sows [53]. The induction of farrowing uses prostaglandins and/or oxytocin, and globally, consumers are becoming more interested in animal industries and how medication and hormones are used [64]. Therefore, the practice of inducing sows is not universally viewed favourably, so induction protocols should be assessed to better suit the developmental needs of the piglet.

### 3.3. Transition into Parturition

Farrowing can be long and problematic for the sow and her piglets. Parity, nutrition and the sow’s environment may influence the farrowing outcome [48,65,66]. Traditional research typically focused on the gestation and lactation periods as separate phases, defined as being before and after farrowing is complete, respectively. More recently, research has redefined the period of change from gestation to lactation as a separate state categorised as the transition period with its own management challenges, including nutritional. A relationship between the time from the last feed to the onset of farrowing and the duration of farrowing has been documented [67]. Specifically, farrowing duration was 3.8 ± 1.5 h, if farrowing started within 3.1 ± 0.34 h of the last meal. However, if farrowing commenced more than 3.13 h after the last feed, there was a decrease in arterial glucose concentrations and an increase in farrowing duration to 9.3 h if farrowing commenced 8 h after the last feed. To our knowledge, only one other study by Gourley et al. [68] has focused on the time from feed to farrow intervals. There was no decrease in farrowing duration recorded, when the feed-to-farrow interval was reduced by more frequent feeding sessions. The difference between the two studies has been suggested to be due to the difference in average farrowing duration and born alive for the study populations. This highlights that although some sows may still farrow within a safe timeframe, with the selection for larger litter sizes farrowing duration is likely to increase as will the possible negative effects of prolonged farrowing duration on stillbirth rate and neonatal viability [52,69,70]. Extended farrowing durations increase the incidence and degree of hypoxic events experienced by piglets [12]. Hypoxia slows the responses of piglets to their environment, increases their chance of being overlaid by the sow and/or reduces their ability to compete for sufficient colostrum and milk, lowering their chance of survival. The findings of Langendijk et al. [52] and Feyera et al. [67] highlighted the importance of developing better sow and piglet management strategies during the transition period. Further research into the mechanisms behind the relationship between the time from the last feed to the onset of farrowing and the farrowing duration is crucial for reducing the impact of farrowing duration on piglet viability.

## 4. Management Strategies for Improving Piglet Survival

### 4.1. Sow-Specific Diets

Extensive research has explored numerous diet compositions for sow gestation and lactation feed which may influence sow and piglet performance [71,72]. However, the use of feed additives by producers is limited, as the associated costs can outweigh the benefit. There are generally three main phases targeted for gestation-specific diets, which differ between gilts and sows, with these being early gestation (days 1–28), mid gestation (days 29–84) and late gestation (days 85–115). Early-gestation diets are targeted at priming the sow for optimal metabolic and endocrine conditions to develop and maintain good-quality embryos and foetuses [73]. Mid gestation feeding is focused on maintenance and maternal body gain, usually met by a gradual increase in feed and energy intake [74]. Late gestation is crucial for foetal and mammary growth and influences the production of colostrum and sow performance throughout lactation [75,76]. Despite the transition period from late gestation into lactation being critical for sow performance and piglet development, there is a paucity of research in this area. The transition from gestation to lactation is traditionally considered to occur, after the sow farrows; however, recent research has put more importance on the lead up to farrowing as a part of the transition period to prime the sow to be in a positive energy state prior to farrowing [67]. Parturition requires large amounts of energy, but sows consume little or no feed immediately prior to or during farrowing. Extended farrowing durations reduce piglet viability and increase stillbirth rates. Recent research documented a significant positive association between the time interval from the last feeding to the onset of farrowing and the farrowing duration [67]. These authors suggested the relationship is directly related to a depleted energy supply if the interval between eating and farrowing is more than 3 h. Others have found that farrowing duration, stillbirths and 24 h mortality are not impacted by increased feeding frequency or amount offered in the 2–3-day prepartum, although there is a decrease in overall PWM [77]. Another contributing factor to farrowing difficulty and extended farrowing durations could be weak muscle tone reducing the effectiveness of muscle contractions, and a decline in blood calcium levels may result in insufficient calcium for optimal myometrial contraction and result in the delayed expulsion of piglets. A deficit in calcium can also reduce the effectiveness of both endogenous and exogenous oxytocin, which also may impair myometrial contractions [78]. There are reports of calcium supplementation reducing stillbirth rates, presumably by reducing the duration of farrowing [77,79]. One method for increasing the calcium mobilisation from bone and the uptake from the gut is by manipulation of the dietary cation-anion differences (DCAD) [80]. Negative-DCAD-transition diets are used extensively in the dairy industry to increase milk production and reduce the occurrence of post-parturient hypocalcaemia [80,81,82]. Negative-DCAD diets contain larger amounts of negatively charged ions which when absorbed into the blood cause a mild acidaemia that promotes parathyroid sensitivity and increased mobilisation of calcium from bone, increased renal vitamin D activation and increased calcium uptake from the intestinal tract. Studies have shown that the effects of feeding negative-DCAD diets for an extensive time prior to calving have minimal negative effects on the cow [83,84]. However, one recent study showed that calves born to cows fed a negative-DCAD diet had a lower BW and an average daily weight gain than calves from cows fed positive-DCAD diets [85]. We are not aware of any published research investigating the feeding of negative-DCAD diets on the sow farrowing duration and the stillbirth rate. However, as sows produce litters of multiple young as opposed to singular young in dairy cows, the same effect may not be clear on the individual offspring. These studies identify the importance of sow nutrition immediately prior to farrowing, both in diet content and feeding frequency, for the optimal farrowing performance.

### 4.2. Interventions at Farrowing

Supervision during farrowing is recommended but often not implemented due to the relatively unpredictable timing of piglet delivery. Behavioural indicators, such as bar biting, pawing and nest building attempts in conjunction with colostrum leakage, are the typical indicators for impending farrowing. Unfortunately, the presentation of these signs varies greatly in degree and timing relative to the onset of piglet delivery [86]. Further, these indicators are reliant on a person being present to observe and are used to monitor their progress. Piglet mortality in the neonatal period is significantly reduced by the presence of staff during the farrowing [63,87]. However, to reduce overall PWM, ongoing supervision for at least three days is recommended [63,88]. Supervision allows the use of practices such as timely manual delivery assistance, drying and rubbing of piglets and fostering techniques [65,88]. The ability of production systems to implement the adequate supervision of farrowing may be constrained by labour costs and staff availability. However, modern technology can be used to supervise farrowing and reduce piglet mortality without increased labour costs. With the increasing interest in smart farming technologies across multiple agricultural sectors, there have been growing numbers of studies conducted to develop and demonstrate the use of technology such as movement sensors that detect postural changes or patterns of behaviour to predict the onset of farrowing. It has been noted, however, that most of these technologies require large amounts of power resulting in a need for frequent battery changes and are currently not ready for applications within commercial production [89,90]. Further, these examples are focused on detecting the onset, but not the process of farrowing, which does not replace the labour needed for the adequate supervision of farrowing. Other promising systems, such as thermal cameras which use the detection of heat differences to notify personnel when a sow has not had a piglet in a set interval, would allow for timely assistance and potentially piglet assistance prior to the onset of hypothermia.

The standard farrowing crate design includes a creep area often with some sources of heat such as heat lamps, mats and/or bedding [91,92]. However, piglets prefer to lie close to the udder of the sow during the first 24 h of life, when the risk of hypothermia is greatest [93]. Piglets naturally adapt their behaviours to reduce heat loss by huddling with littermates and shivering [38]. The intensity at which they are able to shiver is inversely related to the body temperature down to 34 °C, but below this, the shivering intensity does not increase and heat production decreases [38]. The early re-warming of a hypothermic piglet can reverse some of the biological reactions and metabolism restrictions associated with the drop in temperature [38]. In human medicine, warm intravenous fluids are given to hypothermic patients to increase their core temperature [94]. This is standard practice during surgical operations as well as in cases of severe hypothermia [95]. Administering warm fluids (65 °C) to hypothermic dogs has been shown to increase their temperature with no adverse reactions [96,97]. To our knowledge, there has been no published research on the administration of warm fluids to piglets as a treatment for hypothermia. Current applied interventions such as drying and rubbing of piglets have been shown to be highly effective in increasing rectal temperature if applied at birth [98]. However, this requires personnel to be present at birth to apply these techniques within an effective time frame. With litter sizes continuing to increase, improved supervision practices will become necessary to maintain piglet neonatal survival and to reduce PWM.

### 4.3. Management during the First 24 h Post-Partum

Fostering is used to increase piglet access to colostrum, milk and warmth, when these essential resources are limited for a piglet to thrive. Various fostering techniques have been developed, but those commonly used are cross-fostering and the use of nurse sows [99,100,101]. Cross-fostering is a standard practice, as it permits the equalising of piglet numbers per litter, allowing more equitable teat access, generally with the minimal movement within a farrowing house. However, if most sows are producing greater numbers of piglets than their teat capacity, then there are limited options to move piglets to available teats. The implementation of split suckling protocols and the use of nurse sows are becoming more popular to ensure piglets in large litters consume sufficient colostrum and milk [88,102]. Split suckling involves the temporary removal of larger piglets from the udder for a series of milk let down events to provide smaller piglets with opportunities to suckle, allowing all piglets to remain with their birth mother [99]. Unlike split suckling, cross-fostering is the movement of piglets from one litter into a new litter to increase their access to colostrum and milk [88]. The proper implementation of split suckling prior to gut closure requires a greater presence of personnel in a farrowing house than does cross-fostering. To ensure lower viability, piglets get the greatest opportunity to consume sufficient colostrum (250 g minimum) and survive, and further investigations into new piglet management options are required.

One-third of sows do not produce enough colostrum to provide 250 g to each of their piglets [29]. The quality of colostrum available to the piglet, rather than the quantity and the amount of colostrum produced per sow, is often not considered, and it has been shown that colostrum quality has not increased with litter size [103,104]. To offset the gap, many energy and milk supplements have become available, but their effectiveness for supporting low-viability piglets is largely undocumented. The main goal of these products is to supply a “boost” of energy to provide piglets with greater opportunities to suckle and obtain colostrum [105,106]. Studies suggest that low-viability and IUGR piglets have underdeveloped gut function, potentially impairing their ability to digest colostrum and supplementary products efficiently [32,33,34]. If the gut is impaired, regardless of the amount of colostrum or energy supplied, nutrient and immunoglobulin uptake from the gut will be reduced. The development of a supplementary product that not only provides energy but also improves the gut function of piglets would be beneficial. As the litter size continues to increase, the need for more research and development in the management of neonatal low-viability piglets becomes increasingly urgent.

## 5. Conclusions/Summary

Figure 1 summarises various interactions impacting PWM. Low-viability piglets are increasingly the result of pressure to drive economic growth by increasing larger litter sizes. The lack of consistency in the definition of low-viability piglets increases the confusion when comparing methods for identifying low-viability piglets and the efficacy of management strategies to improve their survival. The Danish pig industry has proven that selection can successfully increase litter sizes. However, sows may not be able to supply adequate support immediately following birth to nurse them to weaning. Staff attendance and supervision is critical, but the availability of appropriately trained personnel is often the largest production constraint. To optimise the number of high-quality pigs weaned per sow, solutions and management strategies need to be developed to reduce the occurrence of low-viability piglets and improve their survival and growth to weaning. The findings of this review suggest that the current measures used to define low-viability piglets and the focus of management practices to improve their performance and pre-weaning survival rates require further refinement. This review has highlighted the lack of consistency of definitions for low-viability piglets used within the literature which, in turn, questions the effectiveness of a number of these research outcomes for industry applications.

## Data Availability

Not applicable.
